# Dissemination of evidence based interventions for pediatric sleep disorders – The Niagara project: process and outcomes

**DOI:** 10.1016/j.sleepx.2019.100001

**Published:** 2019-02-07

**Authors:** Wendy A. Hall, Jeff Biletchi, Debbie L. Hunter, Stephanie Lemay, Christine Ou, Lynn Rempel

**Affiliations:** a University of British Columbia School of Nursing, T. 201, 2211 Wesbrook Mall, V6T 2B5, Vancouver, British Columbia, Canada; b Niagara College, 100 Niagara College Boulevard, Welland, Ontario, L3C 7L3, Canada; c Niagara Region Public Health and Emergency Services, Sir Isaac Brock Way, Campbell East, Thorold, L2V 0A2, Ontario, Canada; d Brock University, 1812 Sir Isaac Brock Way, St. Catharines, L2S 3A1, Ontario, Canada

**Keywords:** Pediatric, Sleep, Disorders, Public health, Insomnia

## Abstract

**Background/objective:**

This paper describes evidence-based strategies for the dissemination of empirically supported interventions for infant behavioral sleep problems.

**Methods:**

To identify parents' needs, a survey sampled 1022 parents in the Niagara region about use of health resources, tracking occurred of public health nurses' consultations with parents about infant sleep, and nurses obtained sleep workshop evaluation data from 18 parents. A focus group with 10 participants, a survey of Niagara Region Public Health and Emergency Services (NRPH&ES) employees, and consultations with external stakeholders identified gaps in parents' and infants' care and public health nurses' training needs. We developed solutions by creating evidence-based tools and a program for parents and public health nurses. We implemented and disseminated information via sharing tools on the NRPH&ES website, and workshops for community agencies and public health nurses.

**Results:**

Seventy childhood educators, support workers, and social and public health professionals attended our community workshop. Twenty-three public health nurses attended our training workshop. In guided discussion, nurses evaluated the workshop as addressing gaps in knowledge and enhancing NRPH&ES interventions to manage infants' behavioral sleep problems. Fifteen parents attended a sleep workshop pilot, with seven parents indicating a preference for follow-up telephone support. Fifty individuals attended our oral presentation at the Ontario Public Health Convention.

**Conclusions:**

For next directions, community and other public health agencies want access to our tools and program components. We received a research grant to design, implement, and evaluate sharing tools and program components with community agencies (daycares and childcare centres).

## Highlights

• Behavioral sleep problems are common during infancy.
• Parents identify problems but have limited access to evidence-based care.
• Public health nurses can provide evidence-based support to families.
• Public health nurses can offer community agencies evidence-based resources.

## 1 Introduction

Sleep is an integral part of healthy development for children [1] and a significant determinant of health status that has largely been overlooked [2]. Learning to fall asleep and stay asleep (self-soothing to sleep) constitutes an important developmental milestone in early childhood, which forms a basis for development of emotional self-regulation [3], [4].

Behavioral sleep problems affect approximately 30% of children [5], [6], can persist into later childhood [6], [7], [8], and are linked to infants' inability to self-soothe to sleep [6]. Infant behavioral sleep problems include settling difficulties, night waking, and inadequate sleep duration [9]. Night waking and short sleep duration have been associated with a number of negative outcomes for infants and children. Scher (2005) reported that infants with higher levels of night waking and settling difficulties had significantly higher levels of behavioral difficulties [10]. Infants' night waking, based on actigraphy at 10 months of age, was associated with lower cognitive scores [10]. Hsu (2017) linked frequent infant night waking at six months of age to poorer infant health and more illness episodes at six months [11]. Short sleep duration in infancy predicted a higher propensity for obesity in high birth weight infants [12]. Infants with short nighttime sleep duration trajectories over three, six, 12, and 24 months of age had significantly lower cognitive development and language scores at two years of age [13]. Lower proportions of nighttime sleep (representing sleep consolidation) at 12 months have predicted lower levels of executive functioning for children at two [14] and four years of age [15].

Ongoing infant behavioral sleep problems can also negatively affect parental wellbeing, and quality of parent–child interactions [9]. Parents report anger, depressed mood, and distress about their children's sleep [16], [17], [18]. Their concerns about infants' behavioral sleep problems are a major reason why parents seek assistance from health care providers [9].

Although infant sleep constitutes a public health priority and behavioral sleep problems can easily be managed in community settings [19], there is a lack of accessible, accurate, and evidence-based information about infant sleep for both families and health care providers [8]. Parents' attempts to manage infants' behavioral sleep problems, without adequate education and support, undermine their success, confidence, and capacity to manage sleep problems [20]. Their likelihood of success with improving their children's sleep duration, and sleep quality is enhanced by education about sleep and professional support [20]. Unfortunately Canadian health care providers and professionals link their lack of formal training and knowledge about sleep to diminished confidence in their abilities to provide education and advice about behavioral sleep problems to parents [19], [21]. Canadian health care organizational practices have failed to support provision of adequate training for care providers and funding for accessible services so that parents can prevent and manage infants' behavioral sleep problems [19].

Our paper identifies data supporting the challenges arising from gaps in parents' access to evidence-based education about prevention of and treatment for pediatric sleep disorders (infant behavioral sleep problems) in a regional Canadian public health practice setting. We use data to identify needs and gaps in care and describe tools and our program to address the needs and gaps. In addition, we explain implementation and dissemination of the program and tools and discuss our approaches, and next steps.

## 2 Methods and materials

Our design incorporated evidence-based strategies. We underpinned approaches to managing infants' behavioral sleep problems with research findings indicating ranges of appropriate infant sleep duration at varying developmental stages [22], and ranges of longest sleep period and longest self-regulated sleep period [23], [24]. We provided evidence to support behavioral interventions for pediatric insomnia, eg, bedtime routines, graduated extinction [5], [25], and positive effects of behavioral sleep interventions on maternal–infant relationship quality and child wellbeing [5], [26], [27], [28]. The first author, a Niagara Region Public Health and Emergency Services (NRPH&ES) manager and public health nurses, and a nursing professor collaborated with Strive Niagara (a non-profit organization that supports young parents to achieve secondary education), Healthy Babies Healthy Children (funded by the Ontario Ministry of Child and Youth Services), Infant Mental Health, Baby Friendly Initiative in Ontario (BFI-Ontario), and EarlyON Child and Family Centers in Ontario. Our partnership developed tools and a program to respond to identified needs and gaps in care, and implemented and disseminated the tools and the program.

### 2.1 Site

Niagara Region is an Ontario municipality with a population of approximately 449,000 people [29]. Of that population there are 56,280 couples with children and 22,930 lone-parent families [30]. In 2017, there were 4,047 live births in the Niagara Region [29]. The NRPH&ES serves the entire region and delivers several family-focused programs that include prenatal support, prevention and management of postpartum depression, home visiting for at risk parents, supports for managing parenting challenges, and promotion of optimal child behavior and growth and development for children from birth to six years of age. In 2016, NRPH&ES began development of a Parenting Strategy with an overarching vision and mandate to equip all Niagara Region parents with the knowledge, skills, and resources necessary for their children and families to thrive.

### 2.2 Data collection

NRPH&ES collected data about parents' and infants' health care needs and information about gaps in training and care through a variety of methods.

#### 2.2.1 Parents' and infants' needs

The agency sent a region-wide survey inquiring about use of public health resources to all Niagara residents in the fall of 2017. In total, 2327 participants completed the Niagara Region Survey of whom 1022 self-identified as parents. NRPH&ES also sampled parents who attended their agency offerings, eg the Well Baby Clinic. They tracked nurses' consultations with parents about sleep over three years. They also extracted data from the Public Health Call Manager Program to analyze topics for nurses' telephone interactions with clients (Parent Talk Line) and tracked calls about infant sleep over four years. The nurses identified numbers of families who accessed a parenting class related to sleep over three years. Nurses obtained sleep workshop evaluation data from 18 parent participants.

#### 2.2.2 Gaps in programming and training

NRPH&ES conducted a focus group with 10 NRPH&ES family health team members and consulted with external stakeholders, eg EarlyON (Early Ontario programs for children from birth to six years) Child and Family Center staff, childcare providers, and other public health units (n = 30) about potential gaps in programming to support families with infant behavioral sleep problems. In addition, NRPH&ES surveyed infant-child development service staff, family home visitors from the Healthy Babies/Healthy Children Program, professionals from the Child Health program, and public health nurses about evidence-based information necessary to support interventions with parents managing behavioral sleep problems.

## 3 Results

Results from the NRPH&ES data collection provided information about parents' and infants' needs and gaps in care provision and training.

### 3.1 Parents' and infants' needs

In preliminary NRPH&ES survey results, infant and child sleep was amongst the top six parenting challenges identified by parents (n = 1022). The top three challenges included adjustment to parenting, maternal depression, and maternal self-care. In 2017, NRPH&ES documented 137 individual parent calls focused on infants' behavioral sleep problems. Over three years, NRPH&ES identified 160 in-person consults incorporating infant sleep topics during Well Baby Clinic sessions out of 166 visits (96.4%). NRPH&ES previously offered an infant sleep class (focusing on birth to six months) for parents from their Baby Talk Series (one of a set of four postpartum parenting classes). This infant sleep class had 183 parent participants over three years. Eighteen families who had accessed their birth-to-12 month infant sleep workshop (amongst the top three workshops requested by parents) felt overloaded by the volume of sleep content, which extended from birth to one year of age. None of the parents wanted online learning about infant sleep problems; 72% of parents indicated a preference for a follow-up telephone call from the public health nurse.

### 3.2 Gaps in care provision and training

When public health nurses who facilitated infant sleep classes attended a focus group to provide evaluation feedback, they indicated that parents wanted telephone support from nurses, individualized infant sleep plans, and succinct evidence-based information about helping babies sleep. The nurses linked information gaps for parents to their access of contradictory and non-evidence-based information about infant sleep and behavioral sleep problems, mostly from the internet (including Facebook), family, friends, and primary care providers. The public health nurses identified their need for more training about developmental changes in infant sleep and access to empirical support for behavioral sleep interventions. They indicated that a campaign was necessary to reach more parents and educate community primary care providers about sleep. The NRPH&ES staff (eg, child health nurses) responses indicated the necessity to develop more supports for parents with infant sleep concerns and questions.

### 3.3 Tool and program development

Based on the results, a NRPH&ES Reproductive and Child Health Program public health nurse and a Child Health manager worked with the first author to incorporate elements in tools and program materials from a Canadian-based randomized controlled trial to test infant behavioral sleep interventions [16] and from other empirical sources. A nursing faculty member, who provided evidence about supporting breastfeeding, and interdisciplinary and intersectoral stakeholders (Strive Niagara, Healthy Babies Healthy Children, Infant Mental Health, BFI-Ontario, and EarlyON) critically evaluated plans to develop tools and program materials. We used a Social Sciences and Humanities Research Council Connection Grant to support the development of program materials and tools. The team negotiated tensions about co-sleeping, breastfeeding support, and use of behavioral strategies for managing infants' sleep problems [31], [32]. The team members used empirical evidence to support informed parental decision-making based on a clear understanding of evidence, implications, and consequences [33].

### 3.4 Implementation and dissemination of material

To implement material, the team targeted families of infants from birth to six months of age and infants from six months to a year of age using separate components. For infants between zero and six months, we relayed key sleep messages (eg, infant sleep patterns, promoting good sleep habits, and answers to frequently asked questions [FAQ] about common sleep problems) on the Niagara Region Public Health and Emergency Services website (https://www.niagararegion.ca/health/parenting/infant-sleep.aspx#faq). We advertised the website and information through social media, public health programming, and community organizations. We created a tool, a double-sided birth-to-six month sleep tip sheet, for parents and put it on the website.

For families with infants that were six months of age or older, the team developed a multi-component intervention program that targeted parents, health care providers, and community partners. We created a tool, a double-sided six to12-month sleep tip sheet, and a new sleep workshop to focus on six to 12-month-old infants. Other tools for parents included parent work sheets, for documenting routines over the day and detailing a sleep plan. For the program we put together a training manual, with a review of empirical information about infant sleep, care providers' answers for parents' frequently asked questions, and worksheets for providing nurses' telephone follow-up and support for families. These were introduced in the train-the-trainer workshops. Contact information for the NRPH&ES Parent Talk Line (a telephone information line staffed by public health nurses), which provides parents with additional support, is readily available from the website and products in print.

To disseminate the material, we held a workshop for community stakeholders and care providers (70 early childhood educators, support workers, and social and public health professionals) and a training workshop for 23 NRPH&ES nurses. The first workshop aimed to raise stakeholders' and providers' awareness of the importance of healthy infant sleep and increase knowledge about healthy infant sleep, developmental changes in sleep, links between sleep and attachment, sleep-promoting strategies, the nature of behavioral sleep problems, ways to manage sleep problems, and implications of infant behavioral sleep problems for parents and infants.

The training workshop aimed to educate public health nurses about using the materials we had developed to present workshops, provide supporting evidence for the materials, and provide procedural information for telephone assessment and support (the Parent Talk Line). We focused on strategies and tools for nurses to support families' prevention and management of infant behavioral sleep problems. A consultation between the workshop leader and a mother with a healthy older infant was role-modeled to help train the nurses to deliver information to support families.

### 3.5 Description of pilot outcomes

We evaluated our pilot activities through a variety of approaches. Through guided discussion, the group of 23 NRPH&ES nurses indicated that the training workshop was helpful to address current gaps in nurses' knowledge about developmental changes in infant sleep and to enhance NRPH&ES’ efforts to assist parents to manage infants' behavioral sleep problems. Following the training workshop, we piloted a new sleep workshop with parents of six to 12-month-old infants. Fifteen parents attended the workshop. Parents were given time to complete a sleep plan tool with goals during the workshop and were provided with a daytime routines tracking sheet and sleep tip sheet tools. Parents could opt for telephone follow-up 1–2 weeks after the workshop by the public health nurses who had delivered the workshop. The parent and nurse jointly determined the number of follow-up calls. Seven parents indicated they wanted support and received a single telephone follow-up call.

The team presented the tools and program at the Ontario Public Health Convention (TOPHC) a month following the launch of our campaign. Approximately 50 individuals signed up for our session. A number of community agencies in Niagara Region and individuals from 10 public health units have asked for access to our tools and program, and training to use them effectively. For example, one community agency has already received a presentation to staff about key messaging for infant sleep.

## 4 Discussion

Our team addressed the lack of access to evidence-based interventions to manage behavioral sleep problems in infancy through a number of steps.

In the first step, we accessed data about parents' and infants' needs for infant behavioral sleep interventions and public health and stakeholders' perspectives about gaps in programs and training. We used parent-generated data from a Niagara Region Survey and a NRPH&ES sample of parents who engaged with their programs and public health nurses. The parent data we accessed reflected similar concerns to a survey from Western Canada where 95.8% of parents rated sleep issues as a somewhat or very important topic but 40.7% identified lack of knowledge about available services and programs as a barrier to accessing information and support [34]. Similarly to our data, 62.4% of parents in Western Canada preferred to find support via public health drop-in programs and parenting classes/information sessions [34].

We also accessed data from public health nurses and community stakeholders about program gaps for families trying to manage infant behavioral sleep problems and about evidence-based information required by professionals to support interventions with parents managing behavioral sleep problems. The concerns expressed about gaps in programing and training by public health nurses and community stakeholders reflect general provider views in the literature. Canadian health care providers indicated that a lack of formal training and knowledge about sleep diminished confidence in their abilities to provide education and advice about behavioral sleep problems to parents [19], [21]. A recent study of Canadian providers found that almost one-third of respondents delivered advice for behavioral sleep problems that would worsen the problem [35].

In the second step, we created a coalition of partners interested in using evidence-based approaches to behavioral sleep problems in infancy to address the gaps we had identified in care for infants and parents and training for health providers. In the methods section of the paper, we provided a fraction of the evidence supporting our evidence-based strategies in tool and program development. Research findings and systematic reviews support the efficacy of behavioral sleep interventions for older infants [5], [16], [25]. Our collaborative team critically engaged to develop the tools and program, while supporting informed parental decision-making.

In the third step, we implemented and disseminated the material. We divided infants into two groups (zero to six months and six to 12 months). This was an important distinction because behavioral management strategies, such as controlled comforting, are suitable only for infants aged six months or older [36]. We implemented the material through the two workshops: one for community members and a training workshop for public health nurses. We disseminated the material for younger infants using the NRPH&ES website to relay key information about preventing and managing infant behavioral sleep problems. Our implementation of the infant sleep workshop for older infants included tools for families and nurses, and provided access for nurses to a training manual concisely summarizing evidence and detailed responses to frequently asked questions.

Our pilot results suggest that our implementation and dissemination of our tools and program were effective. The public health nurses who received the training indicated the workshop assisted them to deal with gaps in their access to evidence. Fifteen parents attended our new sleep workshop and seven parents opted to follow-up telephone support from the public health nurse. Although we do not have evaluation data from the parents, the number of parents who sought further support suggests that they are actively engaging with the tools and information. In other studies, parents who felt they received unsatisfactory information or engagement from health care providers were unwilling to seek further help [37]. Our public health conference presentation attracted a large number of attendees. Thus far, 10 public health units in Ontario have requested access to our tools and program. Although we have been successful at reaching some parents and a number of public health units, we cannot precisely indicate numbers of families accessing our tools and program; we suggest that the number of families we are reaching is limited. A survey of parents in Western Canada found that 37% of parents identified a lack of time and 36% of parents indicated lack of childcare as barriers to accessing services and programs to manage infant behavioral sleep problems [34]. Our current approach to dissemination does not significantly reduce those barriers.

In response to the limitations of our approach, NRPH&ES and the first author have identified next steps to improve access to evidence-based interventions for families with infant sleep problems and to demonstrate the impact of the interventions that we have developed. We have received a grant to design, implement, and evaluate a program to share infant sleep strategies and resources with community agencies and care providers so that they can support the parents using their services. We aim to test whether the program results in 1) more families receiving support and 2) enhanced outcomes for parents and infants who have received support. It is important to engage with community agencies, such as daycares and childcare centers, and primary care providers, such as physicians and nurse practitioners, to overcome parents' barriers to accessing help to manage infant behavioral sleep problems, such as lack of time and childcare.

## 5 Conclusion

This paper has highlighted work being undertaken by a Canadian medium-sized regional public health unit, researchers, and community stakeholders to provide evidence-based interventions to manage infants' behavioral sleep problems and to reduce barriers to parents' efforts to manage infants' behavioral sleep problems. Increasing parents' access to evidence-based interventions in settings where they engage with their children and their children's care providers every day should assist parents to prevent and manage infant behavioral sleep problems. Our partnership, including researchers, health care providers and social service agencies, is supporting evidence-based practice changes. Our plan to demonstrate the impact of interventions on the target population is necessary to sustain resource commitments to these practice changes.

## Funding

This work was supported by the Social Sciences and Humanities Research Council of Canada Connection Grant [grant number 611-2017-0008, June 2017].
